# Comparative analysis of mechanical properties of orthodontic aligners produced by different contemporary 3D printers

**DOI:** 10.1111/ocr.12537

**Published:** 2021-10-04

**Authors:** Spiros Zinelis, Nearchos Panayi, Georgios Polychronis, Spyridon N. Papageorgiou, Theodore Eliades

**Affiliations:** ^1^ Department of Biomaterials School of Dentistry National and Kapodistrian University of Athens Athens Greece; ^2^ Private practice Limassol Cyprus; ^3^ Clinic of Orthodontics and Pediatric Dentistry Center of Dental Medicine University of Zurich Zurich Switzerland

**Keywords:** 3D printing, clear aligners, instrumented indentation testing, mechanical properties

## Abstract

**Objective:**

The aim of this study was to compare the mechanical properties of orthodontic aligners among different commercially available 3D printing devices.

**Materials and Methods:**

Five 3D printers (Ka:rv LP 550, Swinwon; “KAR”), (L120, Dazz 3D; “L12”), (MiiCraft 125, Miicraft Jena; “MIC”), (Slash 2, Uniz; “SLS”) and (Pro 95, SprintRay; “PRO”) were used to prepare orthodontic aligners with dental resin (Tera Harz TC‐85DAW, Graphy). The central incisors of each aligner were cut, prepared and evaluated in terms of Martens‐Hardness (HM), indentation‐modulus (E_IT_) and elastic‐index (η_IT_) as per ISO14577‐1:2002. Force‐indentation curves were recorded and differences among printers were checked with generalized linear regressions (alpha=5%).

**Results:**

Statistically significant differences were seen for all mechanical properties (*P* < .05), which were in descending order: HM (N/mm^2^) as median (Interquartile Range [IQR]): SLS 108.5 (106.0‐112.0), L12 103.0 (102.0‐107.0), KAR 101.5 (97.5‐103.0), MIC 100.0 (97.5‐101.5) and PRO 94.0 (93.0‐96.0); E_IT_ (MPa) as mean (Standard Deviation [SD]): SLS 2696.3 (124.7), L12 2627.8 (73.5), MIC 2566.2 (125.1), KAR 2565.0 (130.2) and PRO 2491.2 (53.3); and η_IT_ (%) as median (IQR): SLS 32.8 (32.3‐33.1), L12 31.6 (30.8‐32.3), KAR 31.3 (30.9‐31.9), MIC 30.5 (29.9‐31.2) and PRO 29.5 (29.1‐30.0). Additionally, significant differences existed between liquid crystal display (LCD) and digital light processing (DLP) printers for HM (*P* < .001), E_IT_ (*P* = .002) and η_IT_ (*P* < .001), with aligners from the former having higher values than aligners from the latter printer.

**Conclusion:**

Under the limitations of this study, it may be concluded that the mechanical properties of 3D‐printed orthodontic aligners are dependent on the 3D printer used, and thus, differences in their clinical efficacy are anticipated.

## 1 INTRODUCTION

Orthodontic aligners present a highly aesthetic treatment alternative to fixed appliances which make them exceedingly desirable, especially among the adults.^1^, ^2^ The old‐fashioned indirect fabrication technique involves the production of a series of dental models where the thermoplastic material is shaped accordingly either with applied air pressure or under vacuum.^3^ These translucent sequential positioners mainly made of polyethylene terephthalate glycol (PETG) or polyurethane (PU) are able to displace teeth in an incremental fashion^4^, ^5^ to various degrees of clinical effectiveness.^6^ Despite the increased demand for this treatment option, orthodontic aligners remain till today an expensive, tedious and time‐consuming option, which may discourage patients and clinicians alike.

The introduction of direct three‐dimensional (3D) printing technology presents as a breakthrough that solves this problem by providing a low‐cost treatment that can be provided at the same day within the dental office and bypassing the dental lab. In contrast to the conventional indirect technique, the manufacturing process of the direct 3D‐printed technique circumvents the step of the physical construction of the dental model and the aligner is directly constructed based on electronically stored 3D dental data.^7^, ^8^ The materials used are also quite different with epoxy resins and photopolymers being predominant.^7^ Multiple 3D fabrication systems and processes have been developed and employed for that purpose including stereolithography, fused deposition modelling, direct pellet–fused deposition, selective laser sintering, multi‐jet photo‐cured polymer process or continuous liquid interface production technology.^7^, ^8^ Direct light processing (DLP) and liquid crystal display (LCD) are fast 3D printing processes which utilize a conventional light source applied to the entire photopolymer resin and gains acceptance and preference by the majority of aligner 3D‐printer manufacturers.^7^

However, this “do it yourself” trend, as tempting as it appears to be, lacks scientific data regarding fundamental parameters like the material's mechanical properties as well as biocompatibility.^9^ In the literature, there is vast information about the conventionally used thermoplastic aligners like Invisalign and other clear aligner systems. This is, however, not the case for the directly 3D‐printed appliances, which not only are fabricated by different materials, but the end product might also be affected by the manufacturing process itself as well as the printer's specifications.^10^ Thus, the clinician is found in a strenuous position where one can quickly produce an aligner, but is unaware of its fundamental properties that impact its clinical performance. These include important mechanical and surface properties like stiffness, elastic relaxation, hardness and roughness that not only can influence treatment efficiency, but may be also associated with iatrogenic implications on the patient's health. The first two characteristics are decisive on light continuous tooth force implementation which is important for proper periodontal biological response. In addition, hardness is associated with resistance to abrasive stimuli deriving from the opposing arch while roughness is related to plaque accumulation and discoloration.^11^, ^12^ Thus, it becomes evident that the arbitrary clinical application of the direct 3D‐printed aligners due to lack of knowledge makes imperative the need of material quality control at a wide spectrum.

Therefore, the aim of this research is to investigate 3D‐printed aligners deriving from five different 3D printing devices as far as their mechanical behaviour concerned. The null hypothesis set was that no statistical differences exist in the mechanical properties of 3D‐printed aligners printed with different printers.

## 2 MATERIAL AND METHODS

### 2.1 Sample preparation

Five different 3D printers were included in this study (Table 1). A full arch orthodontic aligner was designed using Deltaface CAD software (Coruo, Limoges, France) with a thickness of 0.35 mm and an offset of 0.05 mm from the teeth. Seven identical full arch orthodontic aligners were manufactured from each printer employing a dental resin indicated for the manufacturing of orthodontic aligners (Tera Harz TC‐85DAW, Graphy, Seoul, Korea) and following the manufacturers’ instructions. The aligners were placed in a vertical printing orientation with the minimum necessary supports. Then the aligners were centrifuged for 3 minutes for the removal of uncured resin and post‐cured for 10 minutes from cervical and incisal sides in a post‐curing unit (Cure M, Graphy, Seoul, Korea) according to the manufacturer's guidelines.

**TABLE 1:** Brand names of 3D printers tested along with their corresponding manufacturer, printing technology, XY resolution, minimum layer thickness and codes (group name) used in this study. All printers are equipped with a source that emits at 405 nm

Code	3D Printer	Manufacturer	Printing Technology	XY resolution (μm)	Minimum layer thickness (μm)
KAR	Ka;rv LP 550	Shinwon Dental, Seoul, Korea	LCD	N/A	25
L12	L120	Dazz 3D, Shenzhen, China	LCD	47	25
MIC	MiiCraft 125	Miicraft, Jena, Germany	DLP	65	N/A
SLS	Slash 2	Uniz, San Diego, CA, US	LCD	49.8	10
PRO	Pro 95	SprintRay, Los Angeles, CA	DLP	N/A	50

Abbreviations: DLP, Digital Light Processing; LCD, Liquid Crystal Display; N/A, Not available.

### 2.2 Instrumented Indentation Testing (IIT)

The four central incisors of each aligner were cut off and embedded in acrylic resin (Verso Cit‐2, Struers), with their occlusal surfaces parallel to the horizontal plane. Then, the samples were ground up to 4000 grit SiC paper under water cooling and polished with a water‐based diamond suspension (NapR1 DiaPro‐Struers) of up to 1 μm in a grinding/polishing machine (Dap‐V, Struers, Ballerup, Denmark). Then the Martens Hardness (HM), the indentation modulus (E_IT_), and the elastic index (η_IT_) were determined employing a universal hardness testing machine (ZHU0.2/Z2.5, Zwick Roell, Ulm, Germany) with a Vickers indenter. Three force indentation curves were recorded for each specimen and the mean value was used to identify the specimen itself. The curves were recorded employing a maximum load of 4.9 N for a 2 seconds contact time. All mechanical properties were measured according to formulas provided by the international standard ISO14577‐1^13^ using a Poisson's ratio of 0.357.^14^

### 2.3 Statistical analysis

The numerical data of HM, E_IT_ and η_IT_ were initially checked for normality visually and with the Shapiro–Wilk test. Descriptive statistics included means with standard deviations (SD) for normal and medians with interquartile ranges (IQR) for skewed data. Non‐normally distributed data were transformed to approximate normality (square for HM and inverse of square for n_IT_). Generalized linear regression models (GLM) were run on the raw or transformed variable as appropriate with robust standard errors to account for multiple measurements within the same aligner. Post hoc pairwise comparisons were run using a Holm‐Sidak correction for multiple testing. Grouped comparisons of printers according to the printer technology (LCD or DLP) were also run with GLM. All analyses were run in Stata SE 14 (StataCorp, College Station, TX) and boxplots in R, with an alpha=5% and an openly provided dataset.^15^

## 3 RESULTS

**FIGURE 1:**
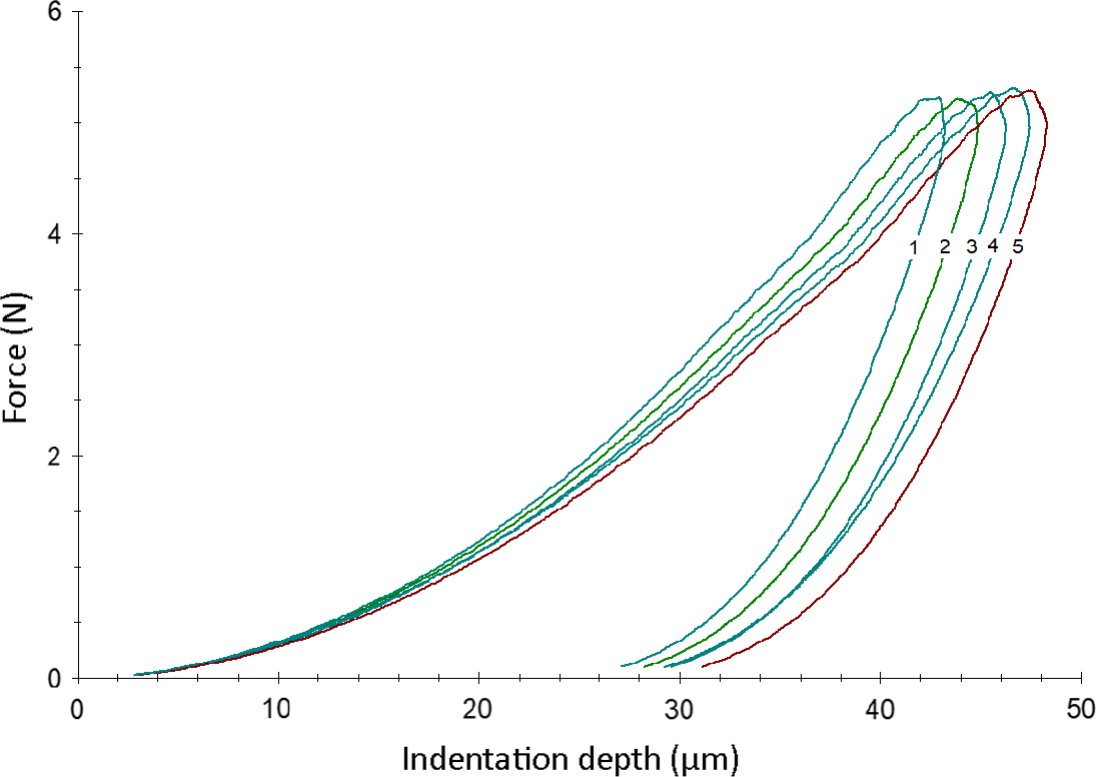
Representative force indentation depth curves for all groups tested. The numerical labels stand for 1:SLS, 2:L12, 3:KAR, 4:MIC and 5: PRO

Figure 1 illustrates representative force‐indentation depth curves from all groups tested. The HM decreases towards deeper indentation depth at maximum force. A steeper unloading curve after maximum indentation has been reached denotes increased E_IT_ values.

**FIGURE 2:**
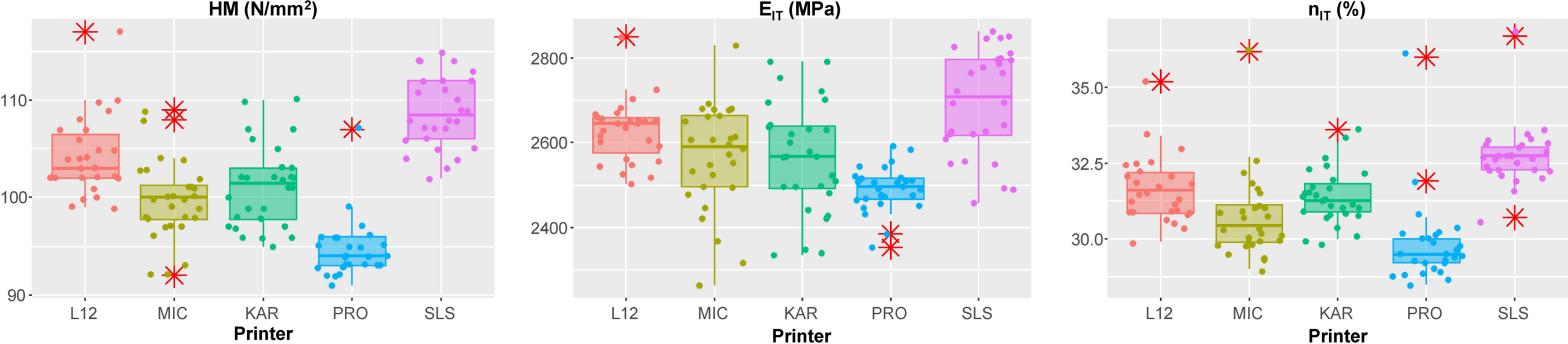
Box plots (ends of boxes: 25^th^ and 75^th^ percentile; line at the median; error bars: quartile plus 1.5 interquartile range; red asterisks: outliers) ΗΜ, E_IT_, and η_ΙΤ_

The measured results for all three outcomes are given in Figure 2. The HM in medians was as follows: in descending order, SLS (108.5 N/mm^2^), L12 (103.0 N/mm^2^), KAR (101.5 N/mm^2^), MIC (100.0 N/mm^2^) and PRO (94.0 N/mm^2^) (Table 2). Posthoc pairwise comparisons (Table 3) indicated three groups: SLS, then L12/KAR/MIC and PRO. The E_IT_ in means was in descending order: SLS (2696.3 MPa), L12 (2627.8 MPa), MIC (2566.2 MPa), KAR (2565.0 MPa) and PRO (2491.2 MPa). Post hoc pairwise comparisons indicated that SLS had higher E_IT_ than all others except L12, the latter having greater E_IT_ than PRO. The η_IT_ in medians was as follows: in descending order, SLS (32.8%), L12 (31.6%), KAR (31.3%), MIC (30.5%) and PRO (29.5%). Post hoc pairwise comparisons indicated again that SLS had higher η_IT_ than all others except L12, the latter (together with KAR) having greater η_IT_ than PRO.

**TABLE 2:** Results of the 3D printers tested for all outcomes

Outcome	L12	MIC	KAR	PRO	SLS	*P* value
HM (N/mm^2^)–median (IQR)	103.0 (102.0, 107.0)	100.0 (97.5, 101.5)	101.5 (97.5, 103.0)	94.0 (93.0, 96.0)	108.5 (106.0, 112.0)	<.001*
E_IT_ (MPa)–mean (SD)	2627.8 (73.5)	2566.2 (125.1)	2565.0 (130.2)	2491.2 (53.3)	2696.3 (124.7)	<.001^†^
n_IT_ (%)–median (IQR)	31.6 (30.8, 32.3)	30.5 (29.9, 31.2)	31.3 (30.9, 31.9)	29.5 (29.1, 30.0)	32.8 (32.3, 33.1)	<.001*

*From generalized linear regression on the transformed variable.

^†^From generalized linear regression on the raw variable.

**TABLE 3:** Post hoc pairwise comparisons after one‐way analysis of variance (HM) or Kruskal–Wallis test (E_IT_ and n_IT_); all with Sidak p‐value corrections for multiple testing

		L12	MIC	KAR	PRO	SLS
HM	L12					
	MIC	0.12				
	KAR	0.52	0.99			
	PRO	<0.001	0.01	<0.001		
	SLS	0.003	<0.001	<0.001	<0.001	
						
		L12	MIC	KAR	PRO	SLS
E_IT_	L12					
	MIC	0.75				
	KAR	0.36	1.00			
	PRO	<0.001	0.44	0.11		
	SLS	0.30	0.04	0.003	<0.001	
						
		L12	MIC	KAR	PRO	SLS
n_IT_	L12					
	MIC	0.37				
	KAR	1.00	0.70			
	PRO	0.004	0.70	0.009		
	SLS	0.08	<0.001	<0.001	<0.001	

## 4 DISCUSSION

The present study evaluated the mechanical properties of orthodontic aligners manufactured via direct process with different 3D printers. Based on the statistically significant differences in all three mechanical properties (HM, E_IT_ and n_IT_) among the printers tested, the null hypothesis must be rejected. 3D printing in dental field is a quickly emerging technology and limited experimental information for the characterization of mechanical properties of orthodontic applications is currently available.^8^, ^9^ Therefore, at the best of our knowledge, there are not similar studies in dental literature and thus no comparison with literature data is feasible.

Interestingly, significant differences were identified for all mechanical properties among the printers tested. Given that all groups share the same resin and identical post‐curing process at the same device, the only source for these differences might be attributed to the 3D printing process of each printer itself. Although the same wavelength (405 nm) was used by all printers, other important parameters, which determine the extent and depth of cure, remain unknown. There is an array of parameters known as “irradiant exposure conditions” which apart from wavelength includes power and exposure time/velocity.^16^, ^17^ The “irradiant exposure conditions” control the extent of polymerization on xy level as well as the depth of cure (z axis), affecting the adherence of curing layer on the previously cured ones and thus the mechanical properties of printed structures in the three axes. Noteworthy is that LCD printers (SLS, L12 and KAR) tend to provide higher HM, E_IT_ and n_IT_ compared to DLP ones (MIC and PRO) (Figure 2). Increased HM and E_IT_ seem to be important from a clinical standpoint, whereas brittle fracture (dueto increased brittleness indicated by higher elastic index) has not been recorded yet as a complication of orthodontic therapy with aligners. These differences in mechanical properties may be attributed to the different technologies used to flash light on the entire layer of resin although both DLP and LCD cure the whole resin layer at once. DLP uses a projector which directs light on selective areas of resin layer by using thousands of minuscule mirrors commonly known as digital micromirror devices while LCD technology uses LCD panels to block off the points that are not to be solidified on each layer.^14^ However, the way that these two different technologies affect the polymerization process should be further investigated.

The range of HM of 3D‐printed groups was found similar to values of clear aligners made of PETG polymer (92 ~ 101 N/mm^2^)^18^ by thermoforming and lower than Invisalign (118 ~ 122 N/mm^2^).^18^, ^19^, ^20^ This means that 3D‐printed aligners are more susceptible to intraoral wear compared to Invisalign ones. Modulus of elasticity of 3D‐printed groups matches to Invisalign (2467 ~ 2616 MPa),^18^, ^19^, ^20^ but is higher than the clear aligner made by thermoforming (2212 ~ 2374 MPa),^18^ a finding which is in accordance with recently published results.^10^ This means that 3D‐printed aligners along with Invisalign appliances may provide higher counter forces under the same strain than the thermoformed ones. Invisalign (40.0 ~ 40.8%) and clear aligner appliances (34.0 ~ 35.9)^18^, ^19^, ^20^ showed a higher elastic index than 3D‐printed appliances implying a more brittle behaviour.

It must be noted here that as per the ISO 14 577 standard, the specimen thickness should be higher than 10 times the maximum indentation depth, which, for this study, should be around 0.40 mm. Specimen thickness in the current study was however 0.35 mm, as this is dictated by the preparation instructions for orthodontic aligners and varying the thickness of the tested specimens would not correspond to the clinical reality, reducing the applicability of this study's results. Likewise, no standard specimens of fixed shape or orientation were printed in this study and 3D printing aligners with irregular morphology might introduce variation in printing precision.^21^ However, as mentioned before, this would result in the specimens not corresponding to aligners used in reality and would have impaired the applicability of this study's findings.

Despite the abovementioned clinical implications of mechanical properties, there is not any evidence that the significant differences in mechanical properties have an effect on clinical efficacy of orthodontic therapy. This requires further analysis employing clinical research. In addition, time‐dependent properties of materials in hand may be more indicative for their clinical efficacy as previous reports have shown an abrupt decrease of orthodontic forces due to relaxation of materials used in manufacturing of orthodontic appliances.^14^, ^19^, ^20^, ^22^, ^23^, ^24^, ^25^ Therefore, experimental research on time‐dependent properties of 3D‐printed structures along with clinical research is a promising field for further research in the field in an effort to deepen our knowledge and optimize the clinical efficacy of these 3D appliances.

## 5 CONCLUSIONS

Within the limitations of this study, the mechanical properties are dependent on 3D printing devices used for the manufacturing of orthodontic aligners.

## CONFLICT OF INTEREST

The authors declare no conflict of interest or financial interest.

## AUTHOR CONTRIBUTION

All authors have contributed to conceptualizing of the work, writing and reviewing the manuscript. In addition, SZ and SNP acquired and analysed data; TE overviewed the coordination of the work.

## Data Availability

The data that support the findings of this study are freely available (10.5281/zenodo.4701075).
